# Should Iliac Wing Screws Be Included in Long Segment Dynamic Stabilization?

**DOI:** 10.7759/cureus.13543

**Published:** 2021-02-24

**Authors:** Ali Fahir Özer, Ahmet Levent Aydın, Mehdi Hekimoğlu, Önder Çerezci, Ahmet T Başak, Ozkan Ates, Tunc Oktenoglu, Mehdi Sasani

**Affiliations:** 1 Neurosurgery, Koç University School of Medicine, Istanbul, TUR; 2 Neurosurgery, American Hospital, Istanbul, TUR; 3 Physical Treatment and Rehabilitation, American Hospital, Istanbul, TUR

**Keywords:** dynesys system, multilevel instability, iliac wing screwing

## Abstract

Background

In this article, clinical satisfaction and radiological results are discussed in a series of patients where the iliac wings participate in dynamic stabilization. Dynamic stabilization is an effective alternative surgical treatment method, especially in clinical pictures that go with pain due to minor instabilities. Practically the unique surgical instrument used in multilevel instabilities is the Dynesys system. The most important drawback of the Dynesys system is that the S1 screws become loose in time. In this article, our aim is to find solution to S1 insufficiency by extension of the system to the iliac wings.

Methods

Nineteen patients (eight females, 11 males) with a mean age of 54.16 were included in the study. Patients had multilevel (level 2 and above) instability, iliac wings were included in the stabilized segments, and Visual Analog Scale (VAS) and Oswestry Disability Index (ODI) were used for patient follow-up.

Results

First year results showed a significant improvement in VAS and ODI. Regarding the complications, infection developed in one patient, loosening in the proximal iliac wing in one patient, and both S1 and iliac proximals in one patient, but no clinical findings were encountered.

Conclusion

When more than two levels of dynamic systems are used in chronic instability, especially in the elderly patients, S1 screws are loosened. In these patients, if the iliac bones are also included in stabilization, this problem is solved successfully. However unfortunately, Dynesys system does not have a screw suitable for the iliac bones.

## Introduction

The concept of dynamic stabilization was defined by Graf in 1991 as an alternative to rigid fixation and fusion surgery in chronic instabilities [1]. The system was later developed by Stoll and Dubois as a Dynesys system and continued to be used thereafter [2]. These systems are based on the principle that the screw is rigid and the rod is dynamic. The spine can perform its physiological movements in all directions in a limited range, to the extent permitted by the dynamic rod.

The next development in dynamic systems was carried out by Von Strempel [3,4]. In this system, the screw head is dynamic and the rod is rigid. Since the rod is rigid, there is a serious limitation in the forward, lateral bending and rotational movements of the spine even at one level. For this reason, it is recommended to use at most for two levels. Hardware complication rates are high in long-term use, even at two levels [5].

These two systems were used together by Kaner et al. [6]. The most physiological results were obtained, matching the movement of a motion segment, and single level complication rates were significantly reduced. Patient satisfaction was reasonable [6-10]. However, this system was not suitable for multilevel use even in this form. A dynamic rod or new design that fits the long level was required. Therefore, the Modular Orthrus System has been developed and is still under development [11-13].

After the Dynesys system, Orthrus system was used in long segment stabilization and multilevel instabilities yielding successful results. Its advantage is to let revision of just the problematic segment instead of the whole system, since it is modular.

However, when you include sacrum to stabilization in long segment stabilizations, although the upper segment is dynamic, the S1 screw is likely to loosen since the whole system is semirigid. For this reason, the biomechanical rules for rigid systems are valid here in dynamic stabilization, and the iliac wings should also be included in dynamic stabilization. In this article, clinical satisfaction and radiological results are discussed in a series of patients where the iliac wings participate in dynamic stabilization.

## Materials and methods

This study includes 19 patients who had multilevel stabilization (eight female, 11 male), the average age is 54.1. Deformity patients without any coronal or sagittal imbalance, patients with multilevel instability due to previous surgery or progressive develop degenerative disc disease, and loosened S1 screw due to osteoporosis were included in the study. The diagnosis, accompanying neurological findings, bone scan results and stabilization levels of the patients are given in Table 1. Dynesys system (Zimmer Spine, Warsaw, IN) was used for all patients. Preoperative and postoperative four-month and one-year clinical and radiological controls of the patients were conducted. Visual analog scale (VAS) and Oswestry scales were used for clinical controls. Radiologically, bone scan, A-P spine radiographs, lumbar CT and MRI were performed for all patients before surgery, and the same examinations were repeated at the 4th month and 1st year controls.

**Table 1:** The diagnosis, accompanying neurological findings, bone scan results and stabilization levels of the patients

	Age	Gender	Previous operation	Complaint	Neurological findings	Diagnosis	Discectomy and instrumentation level	T-score
1	63	M	None	Lumbalgia right sciatalgia	Right L5 radiculopathy	L5-S1 HNP*	L5-S1 discectomy, L3-IW**	2.5
2	50	M	L4-5 discectomy	Left sciatalgia	Right L3-4-5 radiculopathy	L2-3, L4-5, L5-S1 HNP	L5-S1 discectomy, L2-IW	1.8
3	47	M	None	Lumbalgia left sciatalgia	Left L5 radiculopathy	L5-S1 HNP	L5-S1 discectomy, T12-IW	1.7
4	55	F	L5-S1 discectomy	Lumbalgia	Normal	DDD	L3-IW	3
5	41	F	None	Lumbalgia left sciatalgia	Normal	L2-S1 DDD	L2-IW	2
6	58	M	L4-5 discectomy	Lumbalgia right sciatalgia	Right Achilles -	L3-4 deg. Spondylolisthesis, L4-5 recurrence	L4-5 discectomy, L3-IW	2.2
7	33	F	None	Lumbalgia left sciatalgia	Motor loss in the left foot	L4-5 HNP, L3-4, L5-S1 DDD	L4-5 discectomy, L3-IW	3
8	64	M	None	Lumbalgia	Right Laseque 45	S1 screw loosening	L3-4 L4-5 decompression, L3-IW	2
9	58	M	T11-S1 IW stabilization	Lumbar and bilateral leg pain	None	S1 screw loosening	T11-IW	2.5
10	69	F	Previous operation twice	Lumbalgia	None	DDD from L1-2 to L5-S1	T12-IW	3
11	48	F	L4-5 discectomy and dynamic stabilization	Lumbalgia left sciatalgia	Left laseque 45, Left Achilles -	L5-S1 HNP	L4-IW	2.5
12	60	M	L5-S1 discectomy and dynamic stabilization	Lumbalgia right sciatalgia	None	L5-S1 recurrence S1 screw loosening	L5-S1 discectomy, L5-IW	2
13	51	M	None	Lumbalgia right sciatalgia	None	DDD, L3-4 HNP	L3-4 discectomy, L1-IW	2.2
14	50	F	None	Lumbalgia right sciatalgia	Right laseque 30	L5-S1 HNP, L4-5 DDD	L5-S1 discectomy, L4-IW	2.5
15	64	F	None	Lumbalgia left sciatalgia	Left femoral stretch test +	T12-S1 DDD Listhesis at L2-3, L4-5	L4-5 discectomy, T10-IW	3.2
16	59	M	Instrumented before (spinal stenosis)	Claudication	Right EHL 3/5, Bilateral Achilles -	Lumbar spinal stenosis, extensive DDD	Decompression, Bilateral L4, L5-S1 foraminotomy, L1-IW	2.5
17	64	M	Discectomy	Right sciatalgia	Right laseque 20	L3-4, L4-5, L5-S1 spinal stenosis	L3-4 unilateral decompression, L4-5, L5-S1 discectomy, L3-IW	2.2
18	51	F	None	Lumbalgia	Normal	L2-3, L3-4, L4-5, L5-S1 DDD	L2-IW	2.6
19	44	M	L4-5 decompression and L2-5 dynamic stabilization	Lumbalgia	Normal	L2-3, L3-4, L4-5, L5-S1 DDD	L2-IW	1.5

Surgical intervention

**Figure 1:**
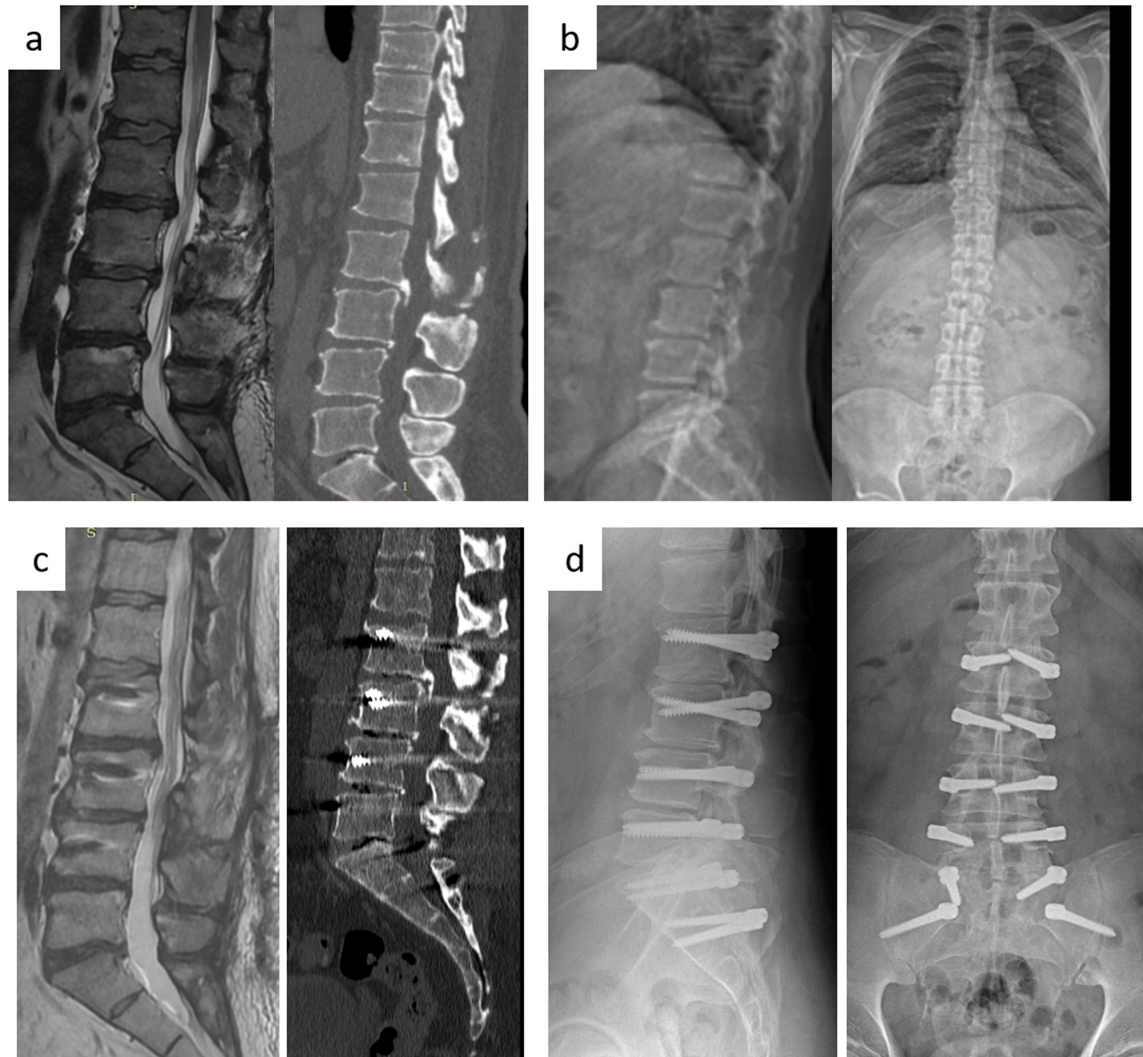
Patient sample a) T2-weighted MR image and CT scan show severe degenerative disc disease in L2-3, L3-4, L4-5 and L5-S1 levels. b) Direct X-ray shows decreased disc height, osteophytes and flat back deformity but no apparent balance problem. c) T2-weighted MR image shows repeated posterior annuluses in every level via microsurgical way and CT scan lateral view shows stabilisation with dynamic system including iliac wings. d) Anterior-posterior (AP) and lateral X-ray.

Decompressive surgery was performed initially in patients with lumbar disc herniation, foraminal or central canal stenosis leading to neurological deficits. Then, after installing transpedicular screws at the previously determined levels, the rod system was tightened and fixed to the screws. Afterwards, the spacers were inserted between the screws (Figure 1). Postoperative flatback deformity was tried to be prevented by giving hyperextension position to the lumbar region of the operating table before inserting the rods.

## Results

There was no change in sagittal and coronal balance in preoperative and postoperative direct X-ray examinations. CT and MR examinations performed on the 4th month and one year after the operation. Four-month CT and MR examinations did not reveal any problems with the screws. In the sense of complications, infection developed in one patient, loosening of the iliac wing screw in one patient, and both S1 and iliac proximals in one patient. Infection was detected at one year control CT and MR examinations.

In infected patients iliac screw and rods were removed. After three months of antibiotic treatment, the iliac screw was reinserted and the rods were inserted to the entire system. No loosening was seen in the follow-up controls.

In patient with proximal screw loosening no symptoms were seen. Apart from this infected patient, a significant improvement was found in life quality scales.

VAS and ODI results of the patients, including these two patients, improved greatly (Table 2). Mean preoperative VAS was 7.16, 4th month postoperative VAS was 3.11 and 12th month postoperative VAS was 1.55.

**Table 2:** VAS and ODI results of the patients VAS: Visual Analog Scale; ODI: Oswestry Disability Index.

	Preop VAS	4-month VAS	12-month VAS	Preop ODI	4-month ODI	12-month ODI
1	7.00	4.00	2.00	58.00	36.00	24.00
2	7.00	3.00	1.00	58.00	38.00	18.00
3	8.00	3.00	1.00	68.00	36.00	24.00
4	6.00	2.00	0.00	64.00	12.00	12.00
5	6.00	4.00	2.00	64.00	24.00	16.00
6	7.00	3.00	2.00	62.00	26.00	16.00
7	8.00	4.00	2.00	92.00	38.00	12.00
8	7.00	2.00	2.00	70.00	26.00	16.00
9	8.00	4.00	1.00	62.00	18.00	12.00
10	7.00	5.00	4.00	70.00	52.00	36.00
11	8.00	4.00	2.00	80.00	12.00	6.00
12	7.00	3.00	1.00	64.00	36.00	12.00
13	7.00	3.00	2.00	56.00	18.00	16.00
14	8.00	2.00	0.00	80.00	16.00	12.00
15	6.00	2.00	2.00	62.00	26.00	12.00
16	8.00	2.00	1.00	56.00	18.00	8.00
17	7.00	3.00	1.00	72.00	18.00	16.00
18	7.00	3.00	2.00	68.00	42.00	24.00
19	8.00	3.00	2.00	56.00	32.00	16.00
Mean	7.16	3.11	1.55	67.00	27.33	16.22

Concerning the ODI scores, preoperative ODI score was 67.00, postop four-month ODI score was 27.33 and 12-month postoperative ODI score was 16.22.

## Discussion

One of the most important points of success in spine surgery lies in the detection of instability before surgery or presumption of postsurgical instability. For surgical success, a good balance between decompression and stabilization must be established. Another important point is to choose the most appropriate method for the benefit of the patient.

There are dozens of articles in the literature that show that the dynamic system is an effective treatment modality for chronic instabilities [14-19]. We think the same.

Di Silvestre used the dynamic system for the first time in multilevel instabilities and reported successful results. Since osteotomy may be required in patients with sagittal and coronal imbalance, dynamic systems are not suitable. However, we believe that it is not correct to perform fusion surgery persistently for deformity patients who do not have balance problems. Dynamic systems can be used easily for this group of patients. Although Di Silvestre does not mention S1 screw failure in his articles, in our own experience, we observed that the possibility of loosening of the S1 screws increases when the dynamic system extends two levels above. This problem also existed in fusion surgery. For this reason, it was tried to be solved by installing an anterior support and extension to the iliac wings. Age and associated osteoporosis are of great importance in screw loosening. However, even if the bone density is acceptable, loosening is still possible, especially for S1 screws. When the literature is reviewed, similar complaints are reported for dynamic systems [20,21].

In spinopelvic fixation, either anterior column support, anterior lumbar interbody fusion (ALIF) or posterior lumbar interbody fusion (PLIF) is required to prevent loosening of the S1 screw. Another technique is descending to the iliac crest [22]. Since we have no chance of interbody fusion in dynamic systems, we decided to put screws on the iliac wings so that the S1 screws do not loosen.

As it is known, the lumbosacral region is under the effect of shear forces, which are 100 N, during bending [23]. McCord et al. developed the concept of "pivot point" for this region for the first time biomechanically [24]. They determined the pivot point as the point where the central axis of the middle osteoligamentous column intersects the line between the last lumbar vertebra and the sacrum. Another important concept in sacral fixation is the three-zone theory defined by O'Brien [25]. Zone I is the vertebral body of S1, which includes the cephalate sacral ala. Zone II includes the bony structure of the caudal sacral ala, the vertebral body of S2, extending to the coccyx, and Zone III is the iliac bones. As the fixation becomes caudal, the ability of fixation to maintain stability increases. Therefore, Zone III is important in terms of stabilization and the resistance against pull out increases in the stabilizations made in this zone. In zone III, the fixation point with the screw placed in the ileum remains in front of the lumbosacral pivot point and increases the stability of the construction. For this reason, iliac screws are designed as long screws to pass the pivot point. However, since no screws of this length are designed in the current Dynesys system, we tried to achieve a stabilization equal to the stabilization provided by Zone I, at least with a screw slightly longer than the length of the S1 screw, using the longest screw in hand. When we evaluate the results, we will be able to say that we have been successful. However, we think that, longer screws would lead to better results.

It should not be forgotten that the condition of the muscles, body mass index and bone density also play an important role in screw loosening in fusion surgery. However, another similar important factor is fusion at three or more levels [26]. Because, as the fusion level increases, the lever arm will extend, and the extended lever arm, which includes the upper lumbar vertebrae during bending at the lumbosacral pivot point, will put excessive load on the S1 screw and will cause loosening. Because of their dynamic properties, we can think that S1 and iliac screws do not have to be loaded as much as rigid screws. However, since it shares the load transfer, it continues to carry load in the anterior column regularly. In this case, even if S1 and iliac screws do not take as much load as in rigid systems, but still they continue to take load continuously. It is a fact that, these systems are semirigid systems. Since the iliac screws are consecutive screw system after S1, they should also play an important role in reducing the load on S1. From this point of view, placing two screws on the iliac wings can create a biomechanically strong construction. When the load distribution is balanced in the long segment, it is very advantageous compared to the short segment and the complication rates are reduced [27].

It should not be forgotten that the Dynesys system is currently used for long segments in the market and there is no alternative. The Dynesys system is a semirigid system. It is more rigid than the physiological motion segment of the spine. For this reason, it is called semirigid. In systems combining dynamic screws and dynamic rods which mimic the motion segment mechanics, the stress on the S1 screw will be less [8,9]. However, even if this goal is achieved, we strongly believe that the iliac screws are still necessary biomechanically.

## Conclusions

As a result, inserting screws into the iliac wings without compromising the basic concepts in multilevel spine stabilization, as in rigid fixation, will significantly reduce the loosening of S1 screws. We believe that stabilization systems including more dynamic systems in the future will be for the benefit of screw loosening problems.
